# Human Papillomavirus Knowledge and Vaccine Acceptability in Jazan Province, Saudi Arabia

**DOI:** 10.3390/vaccines10081337

**Published:** 2022-08-17

**Authors:** Atheer I. Darraj, Alshaymaa M. Arishy, Atheer H. Alshamakhi, Njoud A. Osaysi, Shatha M. Jaafari, Shareefa A. Sumayli, Rawiah Y. Mushari, Abdulaziz H. Alhazmi

**Affiliations:** 1 Faculty of Medicine, Jazan University, Jazan 45142, Saudi Arabia; 2 Medical Research Center, Jazan University, Jazan 45142, Saudi Arabia

**Keywords:** HPV, STD, Jazan, Saudi Arabia, vaccination, knowledge

## Abstract

Background: Human Papillomavirus (HPV) is the most common sexually transmitted infection that affects teens and adults in their early 20 s. Screening and HPV vaccination are important preventive measures to reduce cases of HPV and associated complications. Studies about HPV knowledge and vaccine acceptability are scarce in Saudi Arabia. This study evaluated participants’ knowledge, attitude, and acceptability concerning HPV and the HPV vaccine in Jazan Province, Saudi Arabia. Methods: A cross-sectional study was conducted using a pretested survey that assessed knowledge and attitude toward HPV and the HPV vaccine among the population of Jazan Province, Saudi Arabia. Responses were collected from adults between January and March 2022. Data were analyzed using a *t*-test and chi-square test. Results: We included 569 in the study. Most participants were single (65%), females (83%), with a university-level of education (78%). The recorded knowledge score for all participants was 1.99 (out of 10). About half of the participants denied that HPV is a common sexually transmitted infection. Further, 53% were interested in the HPV vaccine, and 63% of participants acknowledged that the HPV vaccine could prevent warts and cervical cancer. About 30% of the participants opposed the vaccine due to religious reasons. Conclusion: The results of our study highlight the inadequate level of knowledge concerning HPV infection, even among highly educated people. Thus, by promoting the HPV vaccine acceptance and use, awareness can be raised in our community to assure better knowledge and achieve higher protection from this virus and its complications.

## 1. Introduction

Human papillomavirus (HPV) is the most common cause of sexually transmitted infection globally [1]. HPV is also a common cause of cervical cancer, which is reported to be the 4th most common female cancer worldwide [2]. In developing countries, cervical cancer is the leading cause of morbidity and mortality among women [3]. In Saudi Arabia, there is a dearth of data evaluating HPV prevalence and its associated complications. However, Alhamlan et al. conducted a hospital-based cohort between 2013 and 2015 among Saudi women and reported 17% positive cases for HPV among 400 tested samples. HPV genotypes 16 and 18 were mostly reported as they were found to be responsible for more than 90% of cases [3,4,5]. Further, it was previously reported that cervical cancer is not rare. It is considered the 9th female cancer in prevalence, with about 358 cases of cervical cancer reported annually [3,4,5,6].

Despite the existence of a safe and effective vaccine against HPV, there has been an increase in HPV cases and its associated complications in the last few years [1]. Implementing programs to raise awareness of the importance of the vaccine varies worldwide. Still, few studies in our region have been conducted to investigate the impact of public knowledge of HPV [2,7,8,9,10,11,12,13,14]. A few regional and national studies from Saudi Arabia showed that the knowledge of HPV is low [9,10,11,12,13,14]. Further, studies from Arabic countries and the middle east and north Africa regions indicated poor knowledge of HPV and its consequences [15,16], findings that may reflect cultural and religious factors in the region.

The World Health Organization (WHO) estimates that the HPV vaccine will save more than 4 million women’s lives in low- and middle-income countries over the next decade [17]. In this regard, the Saudi Food and Drug Administration (SFDA) has approved the prophylactic HPV vaccinations for females aged 11 to 26 [18]. HPV vaccinations have been added to the routine immunization schedules for females in Saudi National Immunization Schedule, and HPV vaccines (bivalent and quadrivalent vaccines) have been available in Saudi Arabia since 2010 [19,20]. However, there is a lack of studies that assess vaccine effectiveness and safety.

Education plays a crucial role and enhances knowledge about HPV, and studies have revealed that students who belong to health-sciences faculties, including applied medical sciences and medicine, have better knowledge and perception of HPV vaccination, and those with higher education usually have better knowledge of HPV [12].

Studies about HPV prevalence, knowledge, and HPV vaccine acceptance are absent in Jazan province, southwestern Saudi Arabia, which has almost 2 million inhabitants. Therefore, we aimed in this study to assess the level of knowledge of HPV and the acceptability of the HPV vaccine among adult people who reside in Jazan Province.

## 2. Materials and Methods

### 2.1. Context of the Study

This was a cross-sectional investigation conducted between January and March 2022 that targeted the population of Jazan, southwestern Saudi Arabia.

### 2.2. Data Collection Tool

A semi-structured validated questionnaire [21] was completed using Google form to collect data. The questionnaire contained closed-ended questions. It was developed to assess data about the people’s demographics, knowledge, and attitudes about HPV and vaccination. Demographic data included age, gender, education level, social status, and occupation. Knowledge of HPV items covers transmission, symptoms, and vaccination. Items connected to attitudes toward HPV vaccination cover the concern about vaccination. The developed questionnaire’s content and ability to measure items needed to answer the study’s research question were previously tested and scored [21]. Additionally, a pilot study was conducted with 20 participants from the targeted population to test the questions’ clarity and the time needed to complete the interview. The results of the pilot study were excluded from the final analysis.

### 2.3. Data Collection Process

This study targeted Jazan residents, mainly females 18 and older, regarding HPV knowledge and attitude. Data were collected using the English and Arabic language questionnaire that requires acceptance by the participants. The questionnaire was distributed through social media to reach into selected age group. Data were manually verified, entered, and analyzed using the Statistical Package for the Social Sciences (SPSS version 25). The analysis was used to assess the relationship between the variables.

### 2.4. Sampling Procedure

The sample size proposed for this study was calculated to be 384 participants using http://www.raosoft.com/samplesize.html (accessed on 1 November 2021). This calculation was based on the prevalence of knowledge of 50% (because no previous studies have been conducted in Jazan) and a 95% confidence interval, with an error rate of less than 5% in a population of two million. However, we included 569 participants to increase the significant power of this study.

### 2.5. Study Ethics

The ethical approval to conduct the project was granted by the Jazan University Ethics Committee, with approval number REC-43/05/076 on 6 December 2021. We conducted this study following the ethical guidelines of the Helsinki Declaration and the local guidelines of the National Committee of Bioethics, Saudi Arabia. Participants in this study signed a consent to participate before data collection. All collected data were kept confidential and used for only the purpose of research. Also, the questionnaires did not include participants’ personal information or any other identification methods. All the participants were given the right to continue or withdraw at any time from the study.

## 3. Results

### 3.1. Sample Characteristics

Five hundred sixty-nine participants completed the questionnaire. Most participants in the study were female (83%). The mean age of participants was 26 years. Depending on marital status, the majority were single (65%), while 32% were married participants, and the rest were divided into divorced (2%) and widows (1%). Regarding educational level, the majority hold a diploma and bachelor’s (78%), while 19% have a high school level and only 3% hold a master’s and doctorate. More than half participants were students (57%), followed by employed (25%), and the rest were either looking for jobs or retired. These data are summarized in Table 1.

### 3.2. Knowledge of HPV

The total knowledge score percent of our participants is 20%. Half of the participants knew that HPV was a common sexually transmitted infection (49%), and only 38% acknowledged the relationship between cervical cancer and HPV (Table 2). Further, about 11% of the participants denied that the prevalence of HPV in women is highest among women in their 30s, and 33% believe that most people with genital HPV infections are symptomatic. Moreover, 36% agree that the HPV vaccine is available for males and females. About 20% said the HPV vaccine is not licensed for females older than 26 years of age, and 25% believed that men and women who have been diagnosed with HPV should not be given the HPV vaccine. Approximately 12% knew that sexually active adolescents shouldn’t be tested for HPV before receiving HPV vaccination. In Table 3, we sought factors affecting participants’ knowledge of HPV, and the results showed that mainly age, marital status, and job type might affect participants’ knowledge.

### 3.3. Knowledge of HPV in Females

In Table 4, we summarized participants’ knowledge about HPV in females. About 60% of responders agreed that HPV infection is common among females and that females are at risk of HPV infection. Nearly 32% of participants agree on the relation between genital and anal warts and profound physical, emotional, and financial consequences for females. More than half of the participants agreed that most sexually active women have already contracted HPV infections by the age of 26 years, and HPV infections may contribute to anal or head and neck cancers in females (58%).

### 3.4. Attitude towards HPV Vaccination in Females

Half of the participants agreed that males should be given the human papillomavirus vaccine to prevent possible partner HPV infection and its consequences (54%). About 34% of participants thought there is no need to vaccinate males against HPV if females are already vaccinated against HPV (Table 5). About 60% of participants agreed that it is essential to vaccinate females against HPV to prevent them from getting genital and anal warts. In addition, half of the respondents believed that HPV causes too few cancers among females to make vaccination worthwhile. About 33% of participants believed in other ways to manage warts in women rather than the vaccine, and 48% of participants agreed that it is essential to vaccinate females against HPV to prevent males from getting infected with HPV. Only 21%participants disagreed that “it’s too late to vaccinate against HPV if an adolescent male is already sexually active”.

### 3.5. Attitude towards the HPV Vaccine

Table 6 summarizes participants’ responses that measure attitudes toward the HPV vaccine. One-third of participants were worried that the vaccine would encourage riskier or earlier sexual behavior, and 38% felt that the vaccine was too new. About 37% were concerned about the efficacy of the HPV vaccine, and 45% were concerned about vaccine safety. 29% opposed HPV vaccination for moral or religious reasons, and about 47% were unaware of the vaccination availability for both males and females. About 53% were interested in the HPV vaccine for males, and 47% were more comfortable providing the HPV vaccine to males rather than females. About 53% were interested in the HPV vaccine for males, while 47% were more comfortable providing the HPV vaccine to males than females, and 36% were concerned about vaccine cost.

## 4. Discussion

HPV is a common sexually transmitted disease and a common cause of genital warts and cervical cancer [1]. To overcome the burden of HPV, Saudi Arabia has approved the prophylactic HPV vaccine for females aged 11 to 26. Since 2010 the HPV vaccine has been included in females’ routine immunization schedules in the updated Saudi National Immunization Schedule, and the ministry of health is responsible for its delivery and enforcement [18,19]. However, studies are limited data on the healthcare coverage and the effectiveness of the vaccine or cervical cancer screening, and a few studies from central and western regions of Saudi Arabia reported a lack of knowledge regarding HPV and its associated complications [2,5,6,11,12,13]. Participants in the current study, which covered the southwestern region, had poor knowledge of HPV. We found that the percentage score was 20%, and that about 50% of the participants did not agree that HPV is a common sexually transmitted disease. Only 38% believed HPV is a causative agent for cervical cancer (Table 2). As reported by others [2,8,11], we found that older age and a higher level of education are positively associated with a level of knowledge about HPV (Table 2). Despite this poor knowledge among the participants of this study, about half of the respondents in our study were interested in the HPV vaccine and were indifferent about the vaccine’s safety, efficacy, or cost (Table 5 and Table 6). These data are coherent with an earlier study conducted in Riyadh in 2016, where only 37% of the participants refused to receive the vaccine [8]. Further, in a recent national study in Saudi Arabia (2021), about 55% were willing to take the HPV vaccine if offered, and 73% would advise others to take the HPV vaccine [12]. Likewise, about 50% of male medical students in Jeddah were interested in receiving the HPV vaccine [2]. To date, no official programs included HPV education in general or higher schools in Saudi Arabia. All gathered knowledge resulted from the personal efforts of healthcare workers, and this may explain the poor level of knowledge we found in the current study, and as reported by others [2,5,6,11,12,13]. These findings indicate the importance of implementing national educational programs about HPV and vaccine campaigns directed to the public. This action would increase public knowledge of HPV and consequently enhance public attitudes toward the HPV vaccine.

Despite being incorporated into females’ routine immunization schedules in the national immunization schedule in Saudi Arabia [18,19]. HPV vaccine for men remained optional, and a few articles discussed men’s perception and knowledge of the HPV vaccine. However, as previous studies suggested, gender does not seem an important factor in the level of knowledge and HPV vaccine acceptability. For example, Farsi et al. published a study in 2020 in which data were collected from 517 male medical students. Despite the studied population being medical students, about 75% of the male students had heard of HPV, and only 42% had heard of the HPV vaccine, with poor knowledge scores regarding HPV and its vaccine [2]. On the other hand, Altimimi conducted a study in 2020 among female students at Hail university [22]. Likewise, poor knowledge was reported among participants, and better knowledge could be related to a longer duration of education and being students in medical schools [22]. In the current study conducted on a population of Jazan, and participants were mostly females (80%) and students (60%) (Table 1), questions about HPV in males and females were directed to participants. Poor knowledge was also observed, in that only 36% knew that the HPV vaccine is available for males and females (Table 2), an answer affected neither by the level of education nor gender of the participants (Table 3). However, 53% believed that the HPV vaccine should be given to males (Table 6). Thus, we believe that improving male and female students’ HPV knowledge is crucial, especially for those enrolled in medical faculties as future healthcare providers. Moreover, integrating information about HPV vaccines into the curricula of medical schools is mandatory to guarantee better knowledge among this population.

Religion and religious practices are important factors that could affect the knowledge level concerning HPV and acceptance of the HPV vaccine. In our study, about 30% of the participants were opposed to the HPV vaccine for religious reasons (Table 6). It has been reported, by Bodson et al. in a study published in 2017 and conducted in Utah, that those religious young females were under-vaccinated and, more importantly, under-informed about HPV and its vaccine [23]. However, the relationship between religious practice and vaccine attitudes seems more complex, as it was found by Redd et al. that religious tradition is positively associated with HPV vaccine knowledge and attitudes, and adherence to religious instructions could be a protective factor against HPV [24,25]. In Saudi Arabia, Akkour et al. conducted a national study and found that only 9% of the population admitted to having relationships outside current marriage, either as previous marriages or polygamy [12]. Further, they reported that most females did not know that HPV is transmitted sexually (79%) or that it causes genital warts (82%) and cervical cancers (82%) [12]. Thus, we believe that it is important to discuss HPV consequences and the advantages of the HPV vaccine openly with young females, especially in conservative and religious cultures where females do not feel comfortable discussing their sexual behavior [9].

Despite being one of the few studies in Saudi Arabia that discussed knowledge of HPV and the acceptability of its vaccines, our study bears many limitations. The data have been collected by a self-administered online survey, a method with a known reporting bias. However, due to the conservative nature of our community and the relativity of this topic to sexual aspects, we preferred to limit the interaction with participants as most of them were females (83%). Therefore, data collection was actively distributed via an online self-reported questionnaire, and its distribution depended on the authors’ networks. As such, most of the participants were young, students, and female. It would be insightful to ask participants if they have been diagnosed with HPV and received the HPV vaccine or included questions about smoking as an important cervical cancer risk factor. However, we know that national awareness about HPV increased recently, i.e., a few months before conducting this study [26], and the availability of HPV testing is still limited in our province. Thus, it would be helpful to include national data about the number of HPV infections and those who benefited from the vaccine in future research. Despite these limitations, these data will be useful for encouraging the implementation of community education programs for the general population.

## 5. Conclusions

HPV vaccines, in addition to other preventive methods, play a vital role in reducing the number of anogenital cancers and HPV-related conditions. Overall, vaccines’ efficacy and safety profile indicate that they will provide significant health benefits to the population. As more data on the efficacy of these vaccines against HPV-related head and neck cancer becomes available, this study highlights the low level of knowledge among both male and female populations from the southwestern region of Saudi Arabia about HPV infection and its vaccine. The lack of knowledge was reported even among highly educated people since HPV infection is a preventable disease. Recently, health officials have started promising national HPV awareness campaigns to increase public awareness of HPV and the importance of its vaccine. Consequently, we emphasize the importance of educating and raising awareness among women about the risk factors of cervical cancer, as well as the importance of screening programs. A well-designed health education program about cervical cancer and the advantages of screening and vaccination would raise awareness among the Saudi population.

## Figures and Tables

**Table 1:** Demographic data of the participants, n = 569.

Variable	Description	
Sex	n	%
Female	472	83%
Male	97	17%
Age (Years)	Mean	SD
	26	7
Marital Status	n	%
Single	371	65%
Married	182	32%
Divorced	13	2%
Widow	3	1%
Educational Level	n	%
High School	110	19%
Diploma and Bachelor	443	78%
Master and PhD	16	3%
Job	n	%
Student	325	57%
Employed	140	25%
Looking for job	98	17%
Retired	6	1%

**Table 2:** Knowledge of HPV.

Item	True		False		Don’t Know	
HPV is a relatively uncommon sexually transmitted infection.	280	49%	75	13%	214	38%
Almost all cervical cancers are caused by HPV infection.	217	38%	108	19%	244	43%
The incidence of HPV in women is highest among women in their 30s	255	45%	61	11%	253	44%
Most people with genital HPV infections are symptomatic.	185	33%	132	23%	252	44%
Genital warts are caused by the same HPV types that cause cervical cancer.	219	39%	63	11%	287	50%
Sexually active adolescents should be tested for HPV before starting HPV vaccination.	246	43%	66	12%	257	45%
HPV vaccine is not licensed for females older than 26 years of age.	121	21%	122	21%	326	57%
HPV vaccine is available for both males and females.	205	36%	83	15%	281	49%
Men and women diagnosed with HPV should not be given the HPV vaccine.	144	25%	81	14%	344	61%

**Table 3:** Factors that affect knowledge about HPV.

	HPV Is a Relatively Uncommon Sexually Transmitted Infection.		Almost All Cervical Cancers Are Caused by HPV Infection.		The Incidence of HPV in Women Is Highest among Women in Their 30s		Most People with Genital HPV Infections Are Symptomatic.		Genital Warts Are Caused by the Same HPV Types That Cause Cervical Cancer.		Sexually Active Adolescents Should Be Tested for HPV before Starting HPV Vaccination.		HPV Vaccine Is Not Licensed for Females Older than 26 Years of Age.		HPV Vaccine Is Available for Both Males and Females.		Men and Women Who Have Been Diagnosed with HPV Should Not Be Given the HPV Vaccine	
	Correct Answer	*p* Value	Correct Answer	*p* Value	Correct Answer	*p* Value	Correct Answer	*p* Value	Correct Answer	*p* Value	Correct Answer	*p* Value	Correct Answer	*p* Value	Correct Answer	*p* Value	Correct Answer	*p* Value
Sex																		
Female	13%	0.32	37%	0.144	12%	0.114	23%	0.829	12%	0.438	11%	0.52	22%	0.904	36%	0.0969	14%	0.69
Male	15%		44%		5%		23%		8%		13%		20%		35%		16%	
Age in years. median: SD	28:8	0.0001 *	25:6	0.045 *	26:6	0.516	25:5	0.116	26:6	0.002 *	26:6	0.067	26:7	0.109	25:6	0.76	26:6	0.62
Marital status																		
Single	12%	0.0001 *	43%	0.025 *	12%	0.41	26%	0.085	9%	0.013 *	13%	0.107	24%	0.017 *	38%	0.337	14%	0.755
Married	13%		27%		8%		17%		15%		9%		16%		32%		15%	
Divorced	31%		46%		38%		15%		7%		7%		23%		31%		15%	
Widow	33%		33%		0		0		0		0		0		0		0	
Education																		
High School	9%	0.061	37%	0.736	10%	0.982	14%	0.112	14%	0.864	13%	0.458	25%	0.542	30%	0.294	12%	0.586
Diploma and Bachelor	15%		39%		11%		25%		10%		11%		20%		37%		14%	
Master and PhD	0%		31%		13%		36%		12%		25%		31%		44%		25%	
Job																		
Student	12%	0.028 *	43%	0.032 *	11%	0.529	27%	0.082	9%	0.015 *	13%	0.204	22%	0.453	37%	0.954	13%	0.621
Employed	14%		31%		13%		21%		14%		11%		21%		36%		17%	
Looking for job	15%		29%		8%%		15%		11%		9%		20%		34%		14%	
Retired	0		66%		0%		0		0		0		16%		33%		0	

SD: Standard deviation. * Significant in univariate analysis (The alpha criterion for *p*-value was set to 0.05).

**Table 4:** Knowledge of HPV in females.

Item	Don’t Know		Agree		Disagree	
Females are at risk for HPV infection.	173	30%	359	63%	37	7%
HPV infection is common among females.	191	34%	350	62%	28	4%
Genital and anal warts can cause serious physical, emotional, and financial consequences for females.	257	45%	179	32%	133	23%
Nearly all sexually active females have been infected with HPV by age 26.	209	37%	332	58%	28	5%
HPV infections may contribute to anal or head and neck cancers in females.	209	37%	332	58%	28	5%

**Table 5:** Attitudes Toward HPV Vaccination in females.

Item	Don’t Know		Agree		Disagree	
We should vaccinate males against HPV to protect their future partners from cervical cancer and other consequences of HPV	223	39%	307	54%	39	7%
We already vaccinate females against HPV, so there is no need to vaccinate males as well.	258	54%	195	34%	116	20%
It would be important to vaccinate females against HPV to prevent them from getting genital and anal warts	193	34%	357	63%	19	3%
HPV causes too few cancers among females to make it worthwhile to vaccinate them	250	44%	284	50%	35	6%
Vaccinating females doesn’t make sense because genital and anal warts can be managed in other ways.	244	43%	186	33%	139	24%
It would be important to vaccinate females against HPV to prevent males from getting infected with HPV.	253	44%	270	48%	46	8%
It’s too late to vaccinate against HPV if an adolescent female is already sexually active	270	48%	177	31%	122	21%

**Table 6:** Attitudes Toward HPV vaccine in general.

Item	Don’t Know		Agree		Disagree	
I am concerned that vaccination against a sexually transmitted infection may encourage earlier or riskier sexual behavior.	270	47%	177	31%	122	21%
I feel that the vaccine is too new and “hasn’t been around long enough.”	230	40%	217	38%	122	21%
I am concerned about the safety of the HPV vaccine.	256	45%	257	45%	56	10%
I am concerned about the efficacy of the HPV vaccine.	249	44%	213	37%	107	19%
I am opposed to HPV vaccination for moral or religious reasons.	219	38%	165	29%	185	33%
I am unaware that the vaccine is available for both males and females.	257	45%	265	47%	47	8%
I am interested in the HPV vaccine for males.	225	40%	303	53%	41	7%
I am more comfortable providing the HPV vaccine to males than females.	235	41%	268	47%	66	12%
I am concerned about the cost of the vaccine.	262	46%	203	36%	104	18%

## Data Availability

Data are available upon reasonable request from the corresponding author.
